# Early-Onset Autoimmune Disease as a Manifestation of Primary Immunodeficiency

**DOI:** 10.3389/fimmu.2015.00185

**Published:** 2015-04-24

**Authors:** Magda Carneiro-Sampaio, Antonio Coutinho

**Affiliations:** ^1^Department of Pediatrics, Faculdade de Medicina da Universidade de São Paulo, São Paulo, Brazil; ^2^Instituto Gulbenkian de Ciência, Oeiras, Portugal

**Keywords:** primary immunodeficiency, fetal autoimmunity, IPEX, juvenile systemic lupus erythematosus, inflammatory bowel disease

## Abstract

Autoimmune disorders (AID) have been increasingly observed in association with primary immunodeficiencies (PIDs). Here, we discuss the interface between PID and AID, focusing on autoimmune manifestations early in life, which can be diagnostic clues for underlying PIDs. Inflammatory bowel disease in infants and children has been associated with IL-10 and IL-10R deficiencies, chronic granulomatous disease, immunedysregulation-polyendocrinopathy-enteropathy-X-linked syndrome (IPEX), autoinflammatory disorders, and others. Some PIDs have been identified as underlying defects in juvenile systemic lupus erythematosus: C1q-, IgA-, IgM deficiencies, alterations of the IFN-α pathway (in Aicardi–Goutières syndrome due to *TREX1* mutation). IPEX (due to *FOXP3* mutation leading to Treg cell deficiency), usually appearing in the first months of life, was recently observed in miscarried fetuses with hydrops who presented with CD3+ infiltrating lymphocytes in the pancreas. Hemophagocytic lymphohistiocytosis due to perforin deficiency was also identified as a cause of fetal hydrops. In conclusion, PID should be suspected in any infant with signs of autoimmunity after excluding transferred maternal effects, or in children with multiple and/or severe AID.

## Introduction

An increasing number of reports have shown that a substantial proportion of patients with primary immunodeficiencies (PID) develop autoimmune disorders (AID), in addition to increased susceptibility to infections (1, 2). These findings are paradoxical if natural tolerance is thought to result from negative selection, but they are compatible with the notion that physiological autoimmunity is necessary for self-tolerance. In this case, immunodeficiency is the cause of autoimmunity; in fact, they are the two sides of the same coin. Monogenic PIDs may provide unique opportunities to uncover the genetic bases and mechanisms of complex AID, the study of which has already helped to unravel the pathways that drive autoimmunity. For example, studies of immunedysregulation-polyendocrinopathy-enteropathy-X-linked syndrome (IPEX), which is caused by *FOXP3* gene mutations, have critically established the fundamental role of regulatory T cells (Tregs) in natural tolerance, and PID-AID associations have consolidated the notion of dominant tolerance. Thus, contrary to classical notions of “recessive” tolerance (lymphocyte deletion or anergy) that interpret autoimmunity as resulting from excess (auto) reactivity, the PID-AID associations would rather indicate deficiency in mechanisms that physiologically regulate autoreactivities that are always present.

Autoimmune phenomena, however, have also been observed in other types of PIDs, notably in innate immunity defects (1, 2). Although the association between complement deficiencies and systemic lupus erythematosus (SLE) has long been known, it is now recognized that defects in phagocytes and other innate immunity mechanisms also present autoimmunity. In our view, the concept of AID should actually be expanded to include innate disorders that are classified as “autoinflammatory diseases” (3); thus, there is no *a priori* reason, evolutionary or otherwise, to restrict AID to adaptive immunity. On the contrary, all “ridding” functions are potentially aggressive if they escape regulation, particularly in the innate system. This view predicts that AID should eventually be found in invertebrates.

Here, we discuss the interface between primary immunodeficiency and AID, focusing on early-onset (including intrauterine) manifestations. Our aim is to draw attention to AID in early life as being clinical manifestations of underlying PIDs.

## Heterogeneity of PID-AID Associations

As previously noted (1), a handful of PIDs are systematically associated with AID, such that we consider them to be monogenic autoimmune diseases. However, these PIDs are heterogeneous in terms of the age of onset and clinical manifestations (Table 1). IPEX is a life-threatening condition, with some patients developing AID already in fetal life and rarely surviving infancy in the absence of hematopoietic stem cell transplantation (HSCT); type I diabetes, autoimmune enteropathy leading to chronic diarrhea, inflammatory bowel disease (IBD), and cytopenias are the most common autoimmune manifestations; affected infants also present severe allergy and high IgE levels (4, 5). Omenn syndrome (OS), which is also a life-threatening condition, usually appears in the first months after birth through a combination of severe immunodeficiency with allergic hyperinflammation and autoimmunity, with predominant skin involvement and high levels of IgE. OS is also a consequence of loss of regulation because of the highly restricted, oligoclonal CD4 TCR (T-cell receptor) repertoire, resulting from hypomorphic (severe but not null) mutations in critical genes for V-region rearrangement or lymphocyte development (*RAG1, RAG2*, and other SCID-related genes, including *IL7RA, IL2RG, ADA, DCLRE1C*, Ligase IV gene, *CHD7, RMRP, AK2*) (6). Autoimmune polyendocrinopathy candidiasis ectodermal dystrophy (APECED), which is caused by *AIRE* gene defects, is the prototypic disorder of central (thymic) tolerance and is much less severe, with mucocutaneous candidiasis and autoimmune endocrinopathies usually appearing in childhood (7). Mucocutaneous candidiasis is likely of autoimmune origin, caused by auto-antibodies to cytokines involved in anti-fungal immunity (anti-IL-17) (8). Autoimmune lymphoproliferative syndrome (ALPS) represents a group of lymphocyte apoptosis disorders (due to Fas mutations in most cases) with AID targets predominantly in blood cells (2). ALPS usually appears in childhood and may improve with age. Complete C1q deficiency is intimately associated with juvenile SLE development (9). Note that different autoimmune phenotypes are associated with different genetic defects (1), and a major question concerning the mechanisms of disease continues to be the restricted tissue “targeting” of these conditions.

**Table 1:** **Main characteristics of some monogenic PIDs systematically associated to autoimmune manifestations**.

PID	Inheritance	Mutated gene	Main defect	Age of onset	Susceptibility to infection	Main autoimmune manifestations	Other features
IPEX	X-linked	*FOXP3*	Deficiency of regulatory T cells	Neonatal period or first months of life	*Staphylococcus aureus* (sepsis)	Type-1 diabetes mellitus, enteropathy (leading to chronic diarrhea), IBD, hypothyroidism, cytopenias	Failure to thrive, atopic-like eczema, food allergy, high IgE levels, eosinophilia
APECED	AR	*AIRE*	Impaired negative thymic selection of T cells	Childhood	Only chronic mucocutaneous candidiasis	Hypoparathyroidism, hypoadrenalism, hypogonadism, type-1 diabetes mellitus, hepatitis, alopecia, vitiligo	Enamel dysplasia
ALPS	AR, AD	*FAS, FASL, CASP10, CASP8, PRKCD, NRAS*	Defects of lymphocyte apoptosis	Childhood	Normal resistance to infection	Hemolytic anemia, thrombocytopenia, Evans’ syndrome, neutropenia	Lymphadenopathy, splenomegaly, high numbers of TCR αβ double negative T cells in peripheral blood
Omenn syndrome	AR in most cases	Hypomorphic mutations of *RAG1, RAG2, IL7RA, ADA, IL2RG*, others	Severe combined T and B deficiency	Neonatal period or first months of life	A wide variety of infectious agents: *fungi*, viruses, extra and intracellular bacteria, including mycobacteria	Generalized exfoliative erythroderma (the “red baby”), enteropathy, alopecia	Hepatosplenomegaly, eosinophilia, high IgE levels, oligoclonal T cell repertoire
C1q deficiency	AR	*C1QA, C1QB, C1QC*	Complement deficiency	Childhood	Encapsulated bacteria (*Streptococcus pneumoniae, Haemophilus influenzae*)	SLE with severe skin involvement, kidney and CNS also affected in most cases	–
HLH	AR	*PERF1, STX11, STXB2, UNC13D, or UNC18B*	Defects of cytotoxic granules of natural killer cells and cytotoxic T lymphocytes	Infancy, childhood, rarely later onset	Viral infections may be triggering factors; wide variety of infectious agents observed in neutropenic patients	Systemic hyperinflammation with high proinflammatory cytokine response, no classical autoimmune diseases	Fever, hepasplenomegaly, cytopenias, erythrophagocytosis, hypertriglyceridemia, hyperferritinemia
NOMID/CINCA^a^	AD	*NLRP3/CIAS1*	Autoinflammatory disease (cryopyrinopathy)	Neonatal period or first weeks of life	Normal resistance to infections	Exacerbated inflammation, continuous fever, no classical autoimmune diseases	Urticarial cutaneous rash, chronic aseptic neutrophilic meningitis, arthropathy
DIRA^a^	AR	*IL1RN*	Autoinflammatory disease (lack of IL-1 receptor antagonist)	Infancy, early childhood	Normal resistance to infections	Exacerbated inflammation, no classical autoimmune diseases	Pustular dermatitis and multifocal aseptic osteomyelitis

*AD, autosomal dominant; AR, autosomal recessive; ALPS, autoimmune lymphoproliferative syndrome; APECED, autoimmune polyendocrinopathy candidiasis ectodermal dystrophy; CINCA, chronic infantile, neurological, cutaneous and articular syndrome; CNS, central nervous system; DIRA, deficiency of IL-1 receptor antagonist; IBD, inflammatory bowel disease; HLH, hemophagocytic lymphohistiocytosis; IPEX, immunedysregulation-polyendocrinopathy-enteropathy X-linked syndrome; NOMID, neonatal-onset multisystem inflammatory disease; SLE, systemic lupus erythematosus; TCR, T-cell receptor*.

*^a^Autoinflammatory diseases*.

There is another group of PIDs that are strongly associated with AID (more than 20% of affected patients demonstrate autoimmune manifestations), in which two of the most frequent PIDs are included: IgA deficiency and common variable immunodeficiency (CVID) (Table 2) (1, 2); the mechanisms underlying pathogenic autoreactivity are not entirely clear yet.

**Table 2:** **Main characteristics of some PIDs strongly associated to autoimmune manifestations (present in more than 20% of the PID cases)**.

PID	Inheritance	Mutated gene	Main defect	Age of onset	Susceptibility to infections	Autoimmune manifestations	Other features
Wiskott–Aldrich syndrome (WAS)	X-linked	*WAS*	Combined T and B deficiency	Infancy	Encapsulated bacteria (*Streptococcus pneumoniae*), some fungi (*Pneumocystis jiroveci)*	Hemolytic anemia, neutropenia, IgA nephropathy, arthritis, IBD	Bleeding diathesis, thrombocytopenia with small platelets, eczema, high IgE levels
IgA Deficiency	Unknown	Unknown	Antibody deficiency	Childhood, adolescence, adulthood	Recurrent respiratory infections, giardiasis	Hypothyroidism, cytopenias, SLE	Respiratory allergic symptoms
Common variable immunodeficiency (CVID)	Unknown in most cases, AR	Unknown in most cases, *ICOS, CD19, TNFRSF13B* (TACI), *TNFRSF13C* (BAFF-R), others	Antibody deficiency (low IgG, IgA and/or IgM)	Adolescence, adulthood	Recurrent respiratory infections (often pneumonias due to encapsulated bacteria)	Thrombocytopenia, hemolytic anemia, neutropenia, sicca syndrome, rheumatoid arthritis, IBD, SLE, others	Granulomatous disease and splenomegaly in some cases
Chronic granulomatous disease (CGD)	X-linked, AR	*CYBB* (gp91phox), *CYBA* (p22phox),*NCF1* (p47phox),*NCF4* (p40phox)	Deficiency of microbicidal activity of phagocytes	Infancy or childhood in X-linked cases, childhood in AR patients	Catalase-positive bacteria (*Staphylococcus aureus, Burkholderia cepacia*, *Serratia*, others) and fungi (mainly *Aspergillus*), mycobacteria species, including BCGitis	IBD	Formation of granulomas in gastrointestinal, respiratory, and urinary tracts, abscesses in liver, lungs, and even in spleen
C4 deficiency	AR	*C4A, C4B*	Complement deficiency	Childhood	Encapsulated bacteria	SLE	–
C2 deficiency	AR	*C2*	Complement deficiency	Childhood	Encapsulated bacteria	SLE, vasculitis	–

*AR, autosomal recessive; AD, autosomal dominant; IBD, inflammatory bowel disease; SLE, systemic lupus erythematosus*.

There are a few PIDs in which AID is rarely or never observed, even in patients with chronic or recurrent infections (1). Examples of such PIDs include IL-12/IL-23 – IFN-γ axis deficiencies, IRAK-4 deficiency, autosomal dominant Hyper-IgE syndrome (due to *STAT3* mutations leading to deficiency of Th17 cells), and congenital asplenia.

## Fetal Autoimmune Diseases Associated with PIDs

IPEX typically manifests early in life: 45% of patients in the literature presented symptoms in the neonatal period, and there were 11 cases at birth (4). These observations clearly suggest fetal onset of AID, which we have recently demonstrated in two unrelated families (5). In one family, a newborn with intrauterine growth restriction presented at birth with type-1 diabetes, diarrhea, thrombocytopenia, eczematous dermatitis, eosinophilia, high IgE levels, and autoantibodies to pancreatic islet antigens at 4 days of age, with negative maternal serology. In a second family, a different *FOXP3* mutation was identified in two miscarried monochorionic twin male fetuses, who died at 21 weeks of gestation due to hydrops; the fetuses demonstrated CD3+ infiltrating lymphocytes in their pancreases. Both families had a history of repeated miscarriages of males in consecutive generations, as well as premature babies who developed severe fatal diarrhea immediately after birth. Additionally, at the Children’s Hospital of Philadelphia, in the same family, two miscarried fetuses who died due to hydrops were identified as carrying another *FOXP3* mutation (Sara L. Reichert, personal communication, 2014).

We are aware of only limited additional observations of fetal AID: a case of OS at birth due to an IL7Rα deficiency (10) and a few reports on fetal hydrops associated with HLH (hemophagocytic lymphohistiocytosis), including two perforin-deficient twins (11); these cases were described as non-immune hydrops but, as discussed above, we would classify them as AID due to innate immunity mechanisms. Otherwise, in IPEX, autoimmune hemolytic anemia may be the cause of fetal hydrops (5).

In terms of classical views of tolerance, it is surprising that “break of tolerance” can occur when immune system is still immature. Rather than being “broken”, however, we suggest that establishment of tolerance itself was severely compromised in these cases. Thus, given the current knowledge of the age-dependent development of the various lymphocyte classes and effector functions, as well as on the ability of fetuses to produce immune responses (12), it is not surprising that pathological fetal autoimmunity exists and that there is enough time before birth to produce autoantibodies and to develop autoreactive T lymphocytes, which cause destruction of pancreatic beta-cells or other lesions.

Autoimmune diseases in fetal life are, indeed, extremely rare and have always been associated with severe PIDs; hence, all newborns with suspected AID should be investigated for PID.

## PIDs Associated with Juvenile Systemic Lupus Erythematosus

Deficiencies of the early components of the classical complement pathway have long been recognized as strong risk factors for SLE development (13). Complete C1q deficiency may be considered to be a monogenic SLE form, because more than 90% of affected patients develop severe disease, usually beginning early in life. IgA deficiency was identified in 1–6% of SLE patients and is 10–50 times more frequent in SLE than in the general population. We have recently investigated complement and immunoglobulin deficiencies in patients with both juvenile- and adult-onset SLE (9, 14). Among 72 children and adolescents, 16 (22%) were identified with complement [C1q (*n* = 2), C2 (*n* = 2), C4 (*n* = 2)] or immunoglobulin [IgA (*n* = 3), IgM (*n* = 3), IgG2 (*n* = 4)] deficiencies. The only two cases identified before 2 years of age were associated to PIDs: one C1q deficiency of fatal course and a persistent IgM deficiency. In 5 of the 6 cases with complement deficiencies, SLE manifestations began before 10 years of age. In contrast, no complement deficiency was identified among 300 adult-onset SLE, whereas 3 patients had selective IgA deficiency, and 24 showed isolated IgM deficiency (<30 mg/dL), which was repeatedly observed in the follow-up of 5 patients. Although IgM deficiency has not been consistently reported as being a risk factor for SLE development, note the following three details: (a) observations in murine models indicate that IgM autoantibodies may be protective as neutralizers of IgG autoantibodies; (b) deficiencies in serum and/or secretory IgM resulted in elevated levels of lupus-type IgG antibodies; and (c) these clinical cases all support the idea that a lack of IgM production may be relevant to SLE pathophysiology [reviewed in Ref. (9)]. Severe hypogammaglobulinemia was not observed in either juvenile or adult patients in our cohort, but it has been reported that SLE complicates the clinical course of CVID (13). Analyzing gene copy numbers for *C4A* and *C4B* in the same groups, juvenile SLE patients presented lower values than healthy controls and adult-onset SLE patients (Kaline Pereira and Luis Andrade, personal communication).

Aicardi–Goutières syndrome (AGS), another form of early-onset SLE, is seen to be a monogenic form of lupus that brought light to the role of type I interferons and *TREX1* gene mutations in SLE pathophysiology (15). AGS is a genetically determined encephalopathy that phenotypically overlaps with SLE (erythematous skin lesions, oral ulcers, arthritis, thrombocytopenia, leukopenia, positive antinuclear antibodies), and its autosomal-recessive (AR) form is associated with high IFN-α production. Similar to AGS, SLE is associated with alterations in type I interferon metabolism, with upregulation of type I IFN-inducible genes in peripheral blood cells. *TREX1*, which encodes an exonuclease (a DNAse type III), is the most frequently mutated gene in AGS (15), resulting in intracellular accumulation of abnormal single-stranded DNA, which may explain the high levels of IFN-α observed. *TREX1* mutations were observed more frequently (0.5–3%) in a large cohort of SLE patients than in healthy controls, thus supporting the hypothesis of *TREX1* involvement in lupus pathogenesis.

Systemic lupus erythematosus has also been described in some patients with AR Hyper-IgE syndrome caused by *DOCK8* mutations, as well as in some patients with prolidase deficiency, a rare metabolic disease (15). In both monogenic diseases, the patients also presented with atopic-like skin manifestations and high serum IgE levels.

A homozygous missence mutation in the *PRKCD* gene (encoding protein-kinase delta) was recently identified in three SLE children from a consanguineous family (16). It has been observed that Prkcd participates in the deletion of autoreactive B cells, and deficient mice have demonstrated systemic autoimmunity and B cell proliferation.

Interestingly, other PIDs are systematically associated with autoimmune diseases but not with SLE (Table 1). APECED (due to *AIRE* mutations) is of particular interest because hundreds of patients reportedly reached adulthood with several organ-specific AID, but did not develop SLE, suggesting that defective thymic selection of lymphocytes should not represent a relevant pathogenic mechanism (7, 13). Reinforcing this idea, SLE has rarely been described among Down syndrome patients, who demonstrate lower thymic expression of AIRE and are at high risk of developing organ-specific autoimmune diseases, but not lupus (17).

The evidence above that an underlying defect is more common in pediatric SLE than in adult forms suggests that SLE appearance in the first years of life should lead to the suspicion of a PID.

## PIDs in Early-Onset Inflammatory Bowel Disease

Inflammatory bowel disease in infants and young children has been increasingly recognized as being associated with monogenic defects, particularly PIDs such as IL-10 and IL-10R deficiencies, chronic granulomatous disease, Wiskott-Aldrich syndrome, IPEX, IPEX-like syndromes (CD25 deficiency and gain of function in STAT1 signaling), atypical SCID (severe combined immunodeficiency), OS, NEMO (nuclear factor kB essential modulator) deficiency, XIAP (X-linked inhibitor of apoptosis) deficiency, and autoinflammatory disorders (Tables 1 and 2) (4, 18, 19). PIDs associated with “early“ or “very early-onset” IBD (identified before 10 or 6 years of age, respectively) thus represent various defects of adaptive immunity (including regulatory T cells and anti-inflammatory cytokines), innate immunity, as well as neutrophil dysfunctions, but not complement deficiencies (19).

In a multicentric study with 66 IBD patients younger than 5 years of age and different ethnic origins, 16 (24%) cases demonstrated IL-10 or IL-10R deficiencies (18), with precocious symptoms in the first months of life (infantile IBD forms), and perianal disease in most cases. Although age of onset, location, and other characteristics of the intestinal disease, together with clinical and laboratorial features, may indicate an underlying deficiency, phenotypes usually overlap, and genetic testing should be performed not only to identify monogenic defects but also to select appropriate treatment (18, 19). In some entities, including IL-10 signaling defects, HSCT represents a promising therapeutic option and sometimes the only option to ensure patient survival. As a general rule, abnormal susceptibility to infections and the presence of other autoimmune and/or inflammatory phenomena suggest an underlying PID in infants and young children with an IBD picture.

## Most Autoinflammatory Diseases Manifest Early in Life

Monogenic autoinflammatory diseases (MAID) are classified as PIDs (2) and comprise a growing number of recently identified defects, most of which manifest in childhood, and some of which typically manifest in infancy (3, 20). The study of MAID patients has unraveled key regulatory and effector innate immunity pathways and demonstrated that uncontrolled pro-inflammatory cytokine and chemokine responses can be life threatening and cause significant organ damage as do classical AID mediated by autoantibodies and autoreactive T lymphocytes. Familial Mediterranean fever (FMF) and tumor necrosis factor) Receptor Associated Periodic Syndrome (TRAPS) have been identified as the two most frequent entities in various population groups, including a Brazilian one, FMF presenting significant prevalence in eastern Mediterranean populations (3).

Monogenic autoinflammatory diseases characteristically present with non-infectious fever (continuous in some diseases or periodic in others) and systemic and/or disease-specific organ inflammation. The skin, bones, joints, eyes, and central nervous system are the most common affected sites in MAIDs (3). Extensive skin lesions in infants should be a warning sign for MAIDs: generalized urticarial, pustular, erythematonodular or purpuric rash have been described in different diseases. Abdominal pain is also a frequent feature in FMF and TRAPS.

Some MAIDs begin in the first months of life (Table 1). Neonatal-onset multisystem inflammatory disease (NOMID), also called chronic infantile, neurological, cutaneous and articular (CINCA) syndrome, is the most severe form of cryopyrin (a component of il-1β activating complex) associated periodic syndromes (CAPS). CAPS are caused by autosomal dominant gain-of-function mutation in the *NLRP3/CIAS1* gene, and approximately 40% of patients with clinical NOMID/CINCA are negative for germ line mutation by Sanger sequencing. This defect can be diagnosed clinically and it is characterized by continuous low-grade fever, urticarial cutaneous rash, chronic aseptic neutrophilic meningitis, and arthropathy, starting in the first weeks of life (3). If untreated, patients develop permanent organ damage as a consequence of persistent inflammation in the affected organs. IL-1 blocking agents currently represent the most effective treatment for NOMID/CINCA and the other cryopyrinopathies.

Another early-onset MAID is the deficiency of interleukin 1 receptor antagonist (DIRA) (Table 1). Affected patients present with pustular (sparse, in crops or generalized) dermatitis, and multifocal aseptic osteomyelitis, and periostitis; highly elevated levels of acute phase reactants are also observed (3, 21). Fever is often low grade or even absent in DIRA. A recombinant IL-1R antagonist (anakinra) has been successfully used in DIRA patients with rapid resolution of symptoms and normalization of acute phase reactant levels.

Thus, MAID should be suspected in patients with manifestations of excessive inflammatory responses in infancy and childhood.

## Autoimmune Diseases as Warning Signs for PIDs in Infants and Children

After excluding transferred maternal effects, any AID in infants should raise a PID suspicion. Persistent neonatal diabetes mellitus, autoimmune hypothyroidism, and autoimmune cytopenias (primarily thrombocytopenia and hemolytic anemia) may be precocious features of IPEX. Autoimmune cytopenias may also complicate the course of DiGeorge syndrome, combined immunodeficiencies due to *RAG1* gene mutations, and even SCID in babies (10, 22, 23). Regarding AID in young children, an underlying PID (or other monogenic disorder) should be investigated in patients with multiple and/or severe AID, primarily if recurrent or serious infections are also present. In addition to fever of unknown origin (episodic or continuous), extensive skin lesions of various types, as well as persistent inflammation in the joints, bones, eyes, and central nervous system should represent warning signs for MAIDs in early life.

PIDs should also be considered as the underlying defect in cases of fetal hydrops and recurrent miscarriages of unknown cause (5, 11).

## Conflict of Interest Statement

The authors declare that the research was conducted in the absence of any commercial or financial relationships that could be construed as a potential conflict of interest.
